# The prediction of distant metastasis risk for male breast cancer patients based on an interpretable machine learning model

**DOI:** 10.1186/s12911-023-02166-8

**Published:** 2023-04-21

**Authors:** Xuhai Zhao, Cong Jiang

**Affiliations:** Department of Breast Surgery, Harbin Medical University Cancer Hospital, Harbin, China

**Keywords:** Male breast cancer, Machine learning, Nomogram, SEER, Distant metastasis, XGBoost

## Abstract

**Objectives:**

This research was designed to compare the ability of different machine learning (ML) models and nomogram to predict distant metastasis in male breast cancer (MBC) patients and to interpret the optimal ML model by SHapley Additive exPlanations (SHAP) framework.

**Methods:**

Four powerful ML models were developed using data from male breast cancer (MBC) patients in the SEER database between 2010 and 2015 and MBC patients from our hospital between 2010 and 2020. The area under curve (AUC) and Brier score were used to assess the capacity of different models. The Delong test was applied to compare the performance of the models. Univariable and multivariable analysis were conducted using logistic regression.

**Results:**

Of 2351 patients were analyzed; 168 (7.1%) had distant metastasis (M1); 117 (5.0%) had bone metastasis, and 71 (3.0%) had lung metastasis. The median age at diagnosis is 68.0 years old. Most patients did not receive radiotherapy (1723, 73.3%) or chemotherapy (1447, 61.5%). The XGB model was the best ML model for predicting M1 in MBC patients. It showed the largest AUC value in the tenfold cross validation (AUC:0.884; SD:0.02), training (AUC:0.907; 95% CI: 0.899—0.917), testing (AUC:0.827; 95% CI: 0.802—0.857) and external validation (AUC:0.754; 95% CI: 0.739—0.771) sets. It also showed powerful ability in the prediction of bone metastasis (AUC: 0.880, 95% CI: 0.856—0.903 in the training set; AUC: 0.823, 95% CI:0.790—0.848 in the test set; AUC: 0.747, 95% CI: 0.727—0.764 in the external validation set) and lung metastasis (AUC: 0.906, 95% CI: 0.877—0.928 in training set; AUC: 0.859, 95% CI: 0.816—0.891 in the test set; AUC: 0.756, 95% CI: 0.732—0.777 in the external validation set). The AUC value of the XGB model was larger than that of nomogram in the training (0.907 vs 0.802) and external validation (0.754 vs 0.706) sets.

**Conclusions:**

The XGB model is a better predictor of distant metastasis among MBC patients than other ML models and nomogram; furthermore, the XGB model is a powerful model for predicting bone and lung metastasis. Combining with SHAP values, it could help doctors intuitively understand the impact of each variable on outcome.

**Supplementary Information:**

The online version contains supplementary material available at 10.1186/s12911-023-02166-8.

## Introduction

Male breast cancer (MBC) is clinically rare, accounting for approximately 1% of all breast cancers; however, its annual incidence has increased in recent years [1, 2]. Because the incidence of breast cancer in men is much lower than that in women, most breast cancer clinical studies only include women. Therefore, there are few prospective data to guide the clinical treatment of male breast cancer.

Even though the survival rate of breast cancer patients has improved in recent years, patients with distant metastasis still had a worse prognosis, with an overall 5-year survival rate of 27% [3]. Some studies have shown that MBC patients had a worse outcome than females, which could be attributed to a later stage at diagnosis, older age at diagnosis, or a subtype with a poor prognosis, such as triple negative breast cancer (TNBC) [4–7]. Compared with female breast cancer patients who had distant metastasis, MBC patients with distant metastasis showed a higher proportion of simultaneous bone and lung metastasis [8]. However, pairwise analyses of patients with MBC and female breast cancer (FBC) adjusted for stage, age, hormone receptor status, and other variables revealed that MBC patients had a similar or better prognosis than FBC patients [9, 10]. Distant metastasis has a very important impact on the prognosis of MBC patients. Therefore, a tool to predict distant metastasis in MBC patients would be helpful for improving the awareness of cancer prevention among patients and for seeking appropriate treatment in time.

Medical fields have increasingly utilized machine learning (ML) for multiple applications over the past few years, such as the prediction of cancer incidence rates [11], cancer detection [12], cancer survival prediction [13] and bone metastasis risk prediction [14]. However, due to the “black-box” feature of ML models, it is difficult to understand how an ML model predicts an event or why such a feature is vital to outcome. Thus, it is also important to intuitively interpret an ML model so that we can apply the model to clinical work. To solve this disadvantage, the SHapley Additive exPlanations (SHAP) framework was developed in 2017 [15] to help clinicians interpret advanced ML models.

The present study aimed to construct various ML models to predict the distant metastasis risk of MBC patients and to compare their predictive ability of the models with that of a nomogram. Moreover, the SHAP framework was used to identify the best model, which could help provide a more accurate diagnosis and of distant metastasis for male breast cancer patients.

## Materials and methods

### Patient selection

From 2010 to 2015, a total of 2241 MBC (ICD-O-3 8500–8599) patients from SEER database, and a total of 110 MBC patients from our hospital from 2010–2020 were included into this study. The data from the SEER database between 2010 to 2015 included clinical and pathological TNM staging information and could not be distinguished. Therefore, the pathological TNM staging information of patients in our hospital was extracted from postoperative pathological reports according to the 7^th^ AJCC staging system.

Because the SEER database was publicly available, informed consent was not needed. The ethics committee of Harbin Medical University Cancer Hospital approved this study. It was performed in accordance with the World Medical Association Declaration of Helsinki in 1964 and subsequently amended versions. An informed consent form (Titled: Informed consent for secondary utilization of medical history data/biological specimens) was signed by all of the patients from our hospital before the treatment, and a PDF version of this informed consent form is provided in the related files (Chinese and English versions). According to the informed consent form, all the patients consent that the medical history data could be used for scientific research. No biological specimens were used in this study.

The inclusion criteria were as follows: (1) pathologically confirmed MBC (ICD-O-3 8500–8599); (2) unilateral MBC; (3) distant metastasis (including bone, lung, liver and brain metastasis) diagnosed by pathology or imaging examination; and (4) data with AJCC 7^th^ stage.

The exclusion criteria were as follows: (1) the information of distant metastasis is unknown; (2) breast subtype recoded not available/unknown; (3) ER borderline/unknown; and (4) PR borderline/unknown.

**Fig. 1:**
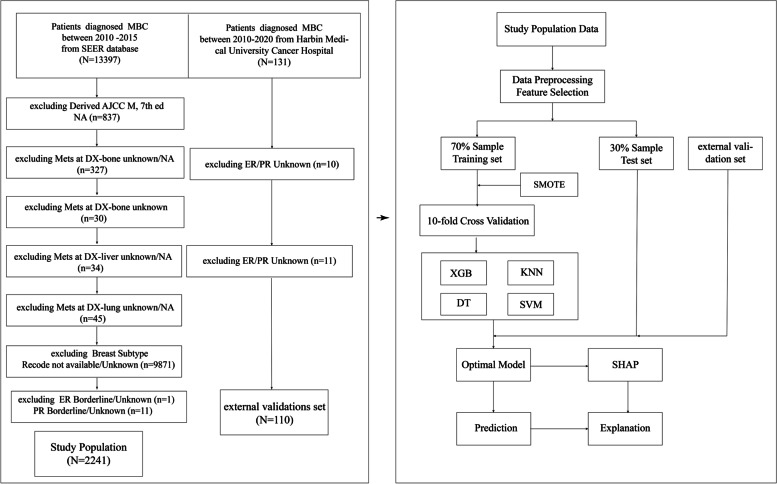
The flow chart of patients selection and the flow chart for the development, evaluation and explanation of models

Figure 1 illustrates the process of selecting patients and developing, evaluating and validating the ML models.

### Feature selection and data preprocessing

Variables with less than 30% missing values were managed by KNNImputer algorithm [16]. Non-hierarchical multiple categorical variables were processed by One-Hot [17]. Fourteen features were selected in this study to predict distant metastasis (M1), including age, laterality, grade, T stage, N stage, radiotherapy, chemotherapy, ER, PR, HER-2, subtype_0 (HR + /HER2-), subtype_1 (HR + /HER2 +), subtype_2 (HR-/HER2-) and subtype_3 (HR-/HER2 +); logistic least absolute shrinkage and selection operator (LASSO) regression was applied to screen the features [18]. Ultimately, age, T stage, N stage, ER status, subtype_0 (HR + /HER2-) and subtype_2 (HR-/HER2-) were selected to develop ML models.

### The development of ML models

A ratio of 7:3 was used for randomly dividing patients into training and test groups. Four powerful ML models were examined in this study, including extreme gradient boosting (XGBoost), k­nearest neighbor (KNN), decision tree (DT) and support vector machine (SVM). In the training set, SMOTE resampling method was applied to address the unbalanced data, and stratified ten-fold CV was applied to prevent overfitting of ML models. A grid search method with ten-fold CV was also applied to optimize the hyperparameters of the ML models. The details are shown in Fig. 1.

### The evaluation of ML models

We assessed the performance of different ML models in the training, testing and external validation set. Models were evaluated and compared according to the area under curve (AUC) [19] and Brier score [20]. Higher AUC values and smaller Brier scores indicate better performance of the ML models.

### The explanation of ML models

To intuitively understand the nature of the ML model with the feature of ‘black-box’, the SHAP framework was introduced into this study to interpret the optimal ML model. Its interpretability performance has been validated in many models [21–23]. The SHAP framework could present global (e.g., summary plot) and local (e.g., force plot) interpretability plots based on SHAP values. The changes in SHAP values reflect the influence of a feature on the outcome.

### Statistical analysis

Categorical variables are shown as proportions, while continuous variables are shown as medians and interquartile ranges (IQRs). The Delong test was applied to compare the performance of different models. The multicollinearity among different variables were tested by multiple linear regression analysis via variance inflation factor (VIF), and a VIF ≤ 5 was considered non-collinearity [24]. Univariable and multivariable analyses were conducted by logistic regression analysis. The nomogram was constructed based on the results of multivariable logistic regression analysis in training set. Discrimination was evaluated by AUC and concordance index (C-index) values of the training, testing and external validation sets. Calibration was assessed by calibration plots. The bootstrapping method was applied for internal validation.

A two-tailed *P* value < 0.05 was considered to indicate statistical significant. R software version 4.1.3, python version 3.9.7 and MedCalc version 19.0.7 were used to carry out all analyses.

## Results

### The clinical and pathological characteristics of MBC patients

A total of 2351 MBC patients were included into this retrospective analysis. The median age was 68 years old. Most patients had Grade 2 (54.7%) and AJCC.T0/Tis/T1 (46.0%). A total of 1306 (55.6%) patients had N0 stage cancer. Most patients did not receive radiotherapy (73.3%) or chemotherapy (61.5%). A total of 2038 (86.7%) patients belonged to the HR + /HER2- subtype. A total of 168 (7.1%) patients had distant metastasis, of whom 117 (5.0%) patients had bone metastasis, and 71 (3.0%) patients had a lung metastasis (Table 1).

**Table 1:** The baseline of all patients

Variables	Overall	train set	test set	external validation set
	*N* = 2351	*n* = 1568	*n* = 673	*n* = 110
**Age** (median [IQR])	68.00 [59.00, 76.00]	68.00 [59.00, 76.00]	68.00 [60.00, 76.00]	66.00 [57.25, 71.75]
**Laterality** (%)				
left	1248 (53.1)	859 (54.8)	338 (50.2)	51 (46.4)
right	1103 (46.9)	709 (45.2)	335 (49.8)	59 (53.6)
**Grade** (%)				
1	256 (10.9)	178 (11.4)	76 (11.3)	2 (1.8)
2	1286 (54.7)	826 (52.7)	362 (53.8)	98 (89.1)
3/4	809 (34.4)	564 (36.0)	235 (34.9)	10 (9.1)
**AJCC.T** (%)				
T0/Tis/T1	1081 (46.0)	694 (44.3)	311 (46.2)	76 (69.1)
T2	970 (41.3)	678 (43.2)	259 (38.5)	33 (30.0)
T2	68 (2.9)	39 (2.5)	28 (4.2)	1 (0.9)
T4/TX	232 (9.9)	157 (10.0)	75 (11.1)	0 (0.0)
**AJCC.N** (%)				
N0	1306 (55.6)	893 (57.0)	359 (53.3)	54 (49.1)
N1	717 (30.5)	460 (29.3)	225 (33.4)	32 (29.1)
N2	201 (8.5)	131 (8.4)	52 (7.7)	18 (16.4)
N3/NX	127 (5.4)	84 (5.4)	37 (5.5)	6 (5.5)
**Radiotherapy** (%)				
no	1723 (73.3)	1146 (73.1)	474 (70.4)	103 (93.6)
yes	628 (26.7)	422 (26.9)	199 (29.6)	7 (6.4)
**Chemotherapy** (%)				
no	1447 (61.5)	979 (62.4)	412 (61.2)	56 (50.9)
yes	904 (38.5)	589 (37.6)	261 (38.8)	54 (49.1)
**Subtype** (%)				
HR( +)/HER2(-)	2038 (86.7)	1355 (86.4)	581 (86.3)	102 (92.7)
HR( +)/HER2( +)	259 (11.0)	173 (11.0)	79 (11.7)	7 (6.4)
HR(-)/HER2(-)	37 (1.6)	27 (1.7)	9 (1.3)	1 (0.9)
HR(-)/HER2( +)	17 (0.7)	13 (0.8)	4 (0.6)	0 (0.0)
**ER** (%)				
negative	58 (2.5)	43 (2.7)	14 (2.1)	1 (0.9)
positive	2293 (97.5)	1525 (97.3)	659 (97.9)	109 (99.1)
**PR** (%)				
negative	205 (8.7)	140 (8.9)	57 (8.5)	8 (7.3)
positive	2146 (91.3)	1428 (91.1)	616 (91.5)	102 (92.7)
**HER2** (%)				
negative	2075 (88.3)	1382 (88.1)	590 (87.7)	103 (93.6)
positive	276 (11.7)	186 (11.9)	83 (12.3)	7 (6.4)
**bone metastasis** (%)				
no	2234 (95.0)	1492 (95.2)	637 (94.7)	105 (95.5)
yes	117 (5.0)	76 (4.8)	36 (5.3)	5 (4.5)
**brain metastasis** (%)				
no	2336 (99.4)	1560 (99.5)	666 (99.0)	110 (100.0)
yes	15 (0.6)	8 (0.5)	7 (1.0)	0 (0.0)
**liver metastasis** (%)				
no	2333 (99.2)	1554 (99.1)	670 (99.6)	109 (99.1)
yes	18 (0.8)	14 (0.9)	3 (0.4)	1 (0.9)
**lung metastasis** (%)				
no	2280 (97.0)	1530 (97.6)	651 (96.7)	99 (90.0)
yes	71 (3.0)	38 (2.4)	22 (3.3)	11 (10.0)
**Distant Metastasis** (%)				
M0	2183 (92.9)	1460 (93.1)	627 (93.2)	96 (87.3)
M1	168 (7.1)	108 (6.9)	46 (6.8)	14 (12.7)

### The performance comparison of different ML models

According to the LASSO regression, the optimal feature number was 6 (Figure S1), including Age, T stage, N stage, ER status, subtype_0 (HR + /HER2-) and subtype_2 (HR-/HER2-). Four ML models were well trained and none of them exhibited overfitting (Figure S2).

**Fig. 2:**
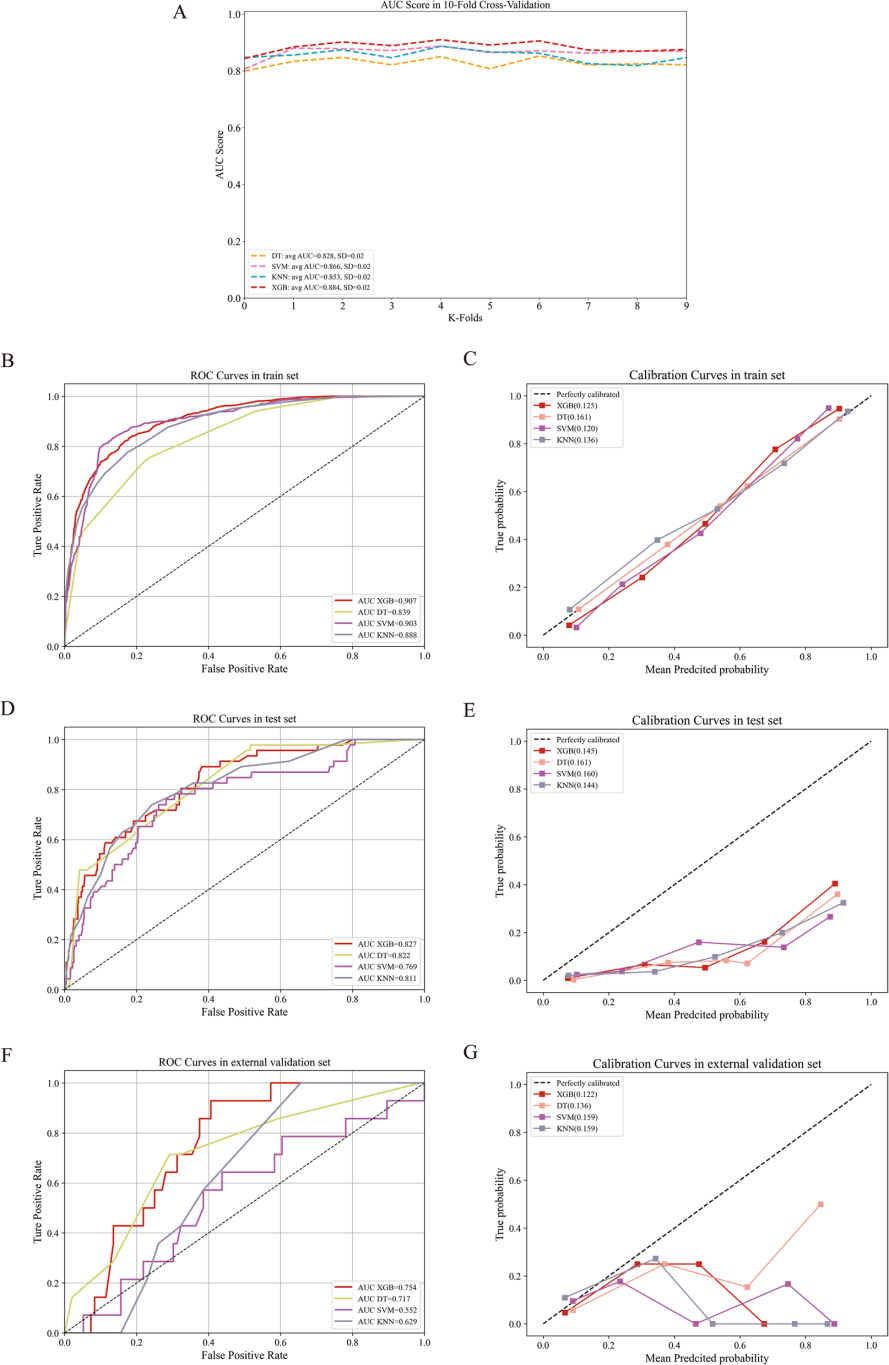
The performance comparison of different ML models. The AUC comparison of different ML models in train set (tenfold cross validation, **A**). The ROC curves of different ML models in train (**B**), test (**D**) and external validation sets (**F**). The calibration curves of different ML models in train (**C**), test (**E**), and external validation sets (**G**)

In the training set, the XGB model showed the largest mean AUC (0.884) by the tenfold CV (Fig. 2A), and the XGB model also demonstrated the biggest AUC (0.907 vs 0.839 vs 0.903 vs 0.888, Fig. 2B) and the second smallest Brier score (0.125 vs. 0.161 vs. 0.120. vs. 0.136, Fig. 2C). In the test set, the XGB model also showed the largest AUC (0.827 vs. 0.822 vs. 0.769 vs. 0.811, Fig. 2D) and the second smallest brier score (0.145 vs. 0.161 vs. 0.160 vs. 0.144, Fig. 2E). In the external validation set, the XGB model also showed the largest AUC (0.754 vs. 0.717 vs. 0.552 vs. 0.629, Fig. 2F) and the smallest Brier score (0.122 vs. 0.136 vs. 0.159 vs. 0.159, Fig. 2G).

To further compare the performance of different ML models, the Delong test was performed. In the training set, the AUC value of the XGB model was significantly larger than that of the DT and KNN models (*p* < 0.05, Table 2). In the test set, no significant difference was observed between the XGB model and other models (*p* > 0.05, Table 2). In the external validation set, the AUC value of the XGB model was significantly larger than that of KNN and SVM models (*p* < 0.05, Table 2).

**Table 2:** The AUC comparison of different ML models in different sets

ML models	Difference of AUC	S. E	95% CI	Z statistic	*p*
**Train set**					
DT ~ KNN	0.0500	0.00551	0.0391—0.0608	9.059	< 0.0001
DT ~ SVM	0.0649	0.00629	0.0526—0.0772	10.321	< 0.0001
DT ~ XGB	0.0689	0.00433	0.0605—0.0774	15.940	< 0.0001
KNN ~ SVM	0.0150	0.00462	0.00591—0.0240	3.241	0.0012
KNN ~ XGB	0.0190	0.00356	0.0120—0.0260	5.339	< 0.0001
SVM ~ XGB	0.00404	0.00388	-0.00355—0.0116	1.043	0.2970
**Test set**					
DT ~ KNN	0.0105	0.0267	-0.0418—0.0627	0.392	0.6952
DT ~ SVM	0.0528	0.0352	-0.0161—0.122	1.503	0.1328
DT ~ XGB	0.00503	0.0160	-0.0263—0.0363	0.315	0.7529
KNN ~ SVM	0.0424	0.0306	-0.0175—0.102	1.386	0.1656
KNN ~ XGB	0.0155	0.0219	-0.0275—0.0585	0.706	0.4801
SVM ~ XGB	0.0579	0.0317	-0.00421—0.120	1.827	0.0677
**External validation set**					
DT ~ KNN	0.0882	0.0836	-0.0757—0.252	1.054	0.2917
DT ~ SVM	0.165	0.120	-0.0695—0.399	1.378	0.1681
DT ~ XGB	0.0368	0.0526	-0.0663—0.140	0.700	0.4838
KNN ~ SVM	0.0766	0.0978	-0.115—0.268	0.784	0.4332
KNN ~ XGB	0.125	0.0616	0.00434—0.246	2.030	0.0423
SVM ~ XGB	0.202	0.0789	0.0471—0.356	2.557	0.0106

Although no significant AUC difference was observed in the test set, which could be attributed to limited Data, the XGB model still showed better performance in the training and external validation sets. Therefore, the XGB model was selected as the optimal ML model for predicting distant metastasis risk in MBC patients.

### The development of nomogram

In the training set, univariable and multivariable logistic regression analyses were applied to explore the independent risk factors for the construction of the nomogram. In the univariable logistic regression analysis, age, grade, AJCC.T, AJCC.N, chemotherapy, subtype, ER, PR and HER-2 were significantly correlated with M1 (*p* < 0.05, Table S1). Then, the multicollinearity among these parameters was tested. Subtype was excluded from multivariate analysis because of a VIF value > 5, and other variables were incorporated. The results of multivariable logistic regression analysis demonstrated that patients with younger age, G3, T3/T4/TX, N ( +) or ER negative status had a higher risk of distant metastasis (*p* < 0.05, Table S1).

Characteristics with *p* < 0.05 in multivariable logistic regression analysis of the training set were incorporated to develop the nomogram (Figure S3A). The C-index for distant metastasis prediction were 0.802 in the training set (Figure S3B), 0.838 in the test set (Figure S3D) and 0.706 in validation set (Figure S3F). Similar results (0.790, 0.838 and 0.701, respectively) were observed when bootstrapping was utilized for internal validation. The distant metastasis prediction was highly consistent with the actual observations in the training set (Figure S3C). However, distant metastasis prediction was not in good agreement with actual observations in the test (Figure S3E) and external validation (Figure S3G) sets.

### The performance comparison of XGB model and nomogram

For a more detailed assessment of the performance of the XGB model, the predictive performance was compared between XGB model and nomogram.

The AUC value of the XGB model was larger than that of the nomogram in the training (0.907 vs 0.802) and external validation (0.754 vs 0.706) sets. The AUC value of XGB model was slightly lower than that of the nomogram in the test validation set (0.827 vs 0.838). In addition, the Z statistic of the XGB model was greater than that of the nomogram in the training (77.248 vs 13.029), testing (10.901 vs 9.764) and external validation (4.915 vs 3.556) sets (Table 3). Therefore, the predictive performance of XGB is better than that of the nomogram.

**Table 3:** The AUC comparison of XGB model and nomogram (based on multivariable logistic regression analysis) in different sets

ML models	AUC	S. E	95% CI	Z statistic	*p*
**Train set**					
XGB	0.907	0.00528	0.896—0.918	77.248	< 0.0001
nomogram	0.802	0.0232	0.782—0.822	13.029	< 0.0001
**Test set**					
XGB	0.827	0.0299	0.796—0.855	10.910	< 0.0001
nomogram	0.838	0.0346	0.808—0.865	9.764	< 0.0001
**External validation set**					
XGB	0.754	0.0517	0.663—0.831	4.915	< 0.0001
nomogram	0.706	0.0579	0.611—0.789	3.556	0.0004

### The prediction of bone and lung metastasis based on the XGB model

**Fig. 3:**
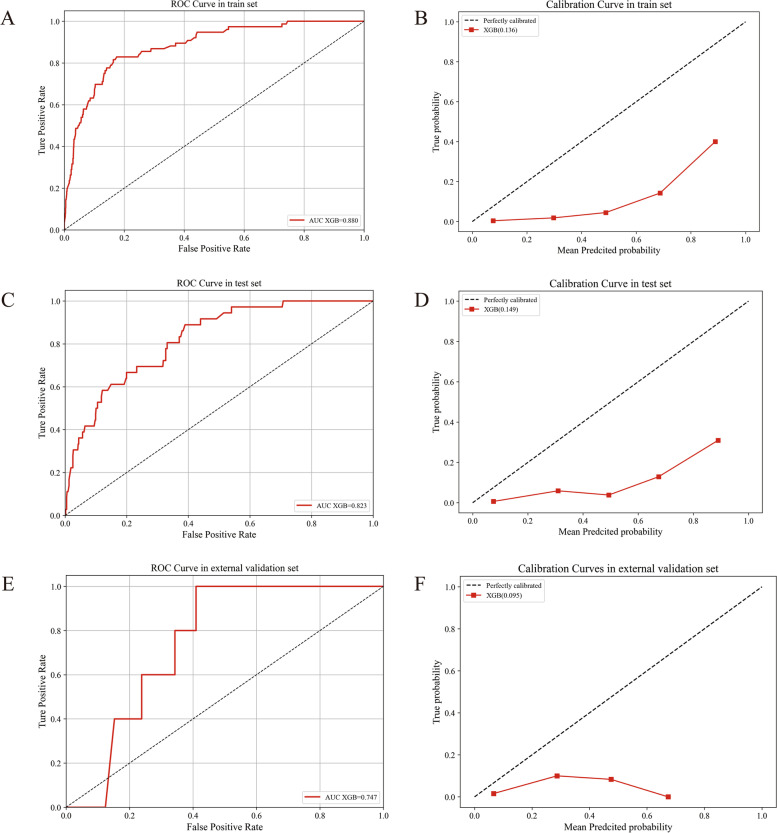
The prediction of bone metastasis based on XGBoost model. The ROC curves of XGBoost model in train (**A**), test (**C**) and external validation sets (**E**). The calibrations of XGBoost model in train (**B**), test (**D**) and external validation sets (**F**)

**Fig. 4:**
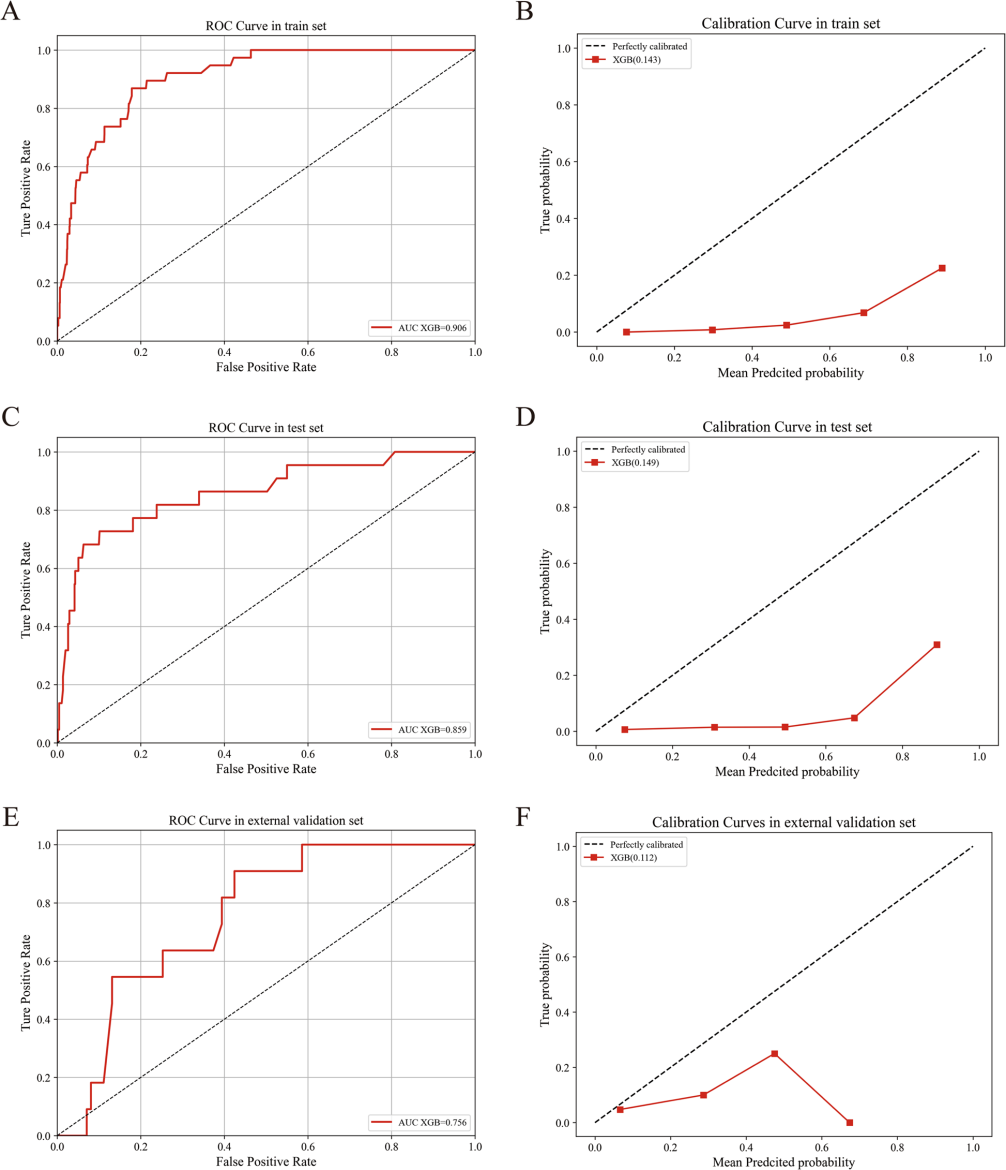
The prediction of lung metastasis based on XGBoost model. The ROC curves of XGBoost model in train (**A**), test (**C**) and external validation sets (**E**). The calibrations of XGBoost model in train (**B**), test (**D**) and external validation sets (**F**)

Based on the above results, the XGB model showed the best predictive ability. The two most common distant metastasis organs were bone and lung [25]. Therefore, we further predicted the risk of bone and lung metastasis for male breast cancer patients based on XGB model. For the prediction of bone metastasis, the XGB model also showed a high AUC value (0.880, 0.823 and 0.747) and a low Brier score (0.136, 0.149 and 0.095) in the training, testing and external validation sets, respectively (Fig. 3). For the prediction of lung metastasis, the XGB model also showed a high AUC (0.906, 0.859 and 0.756) and a low Brier score (0.143, 0.149 and 0.112) in the training, testing and external validation sets, respectively (Fig. 4).

### The interpretability of the XGB model

**Fig. 5:**
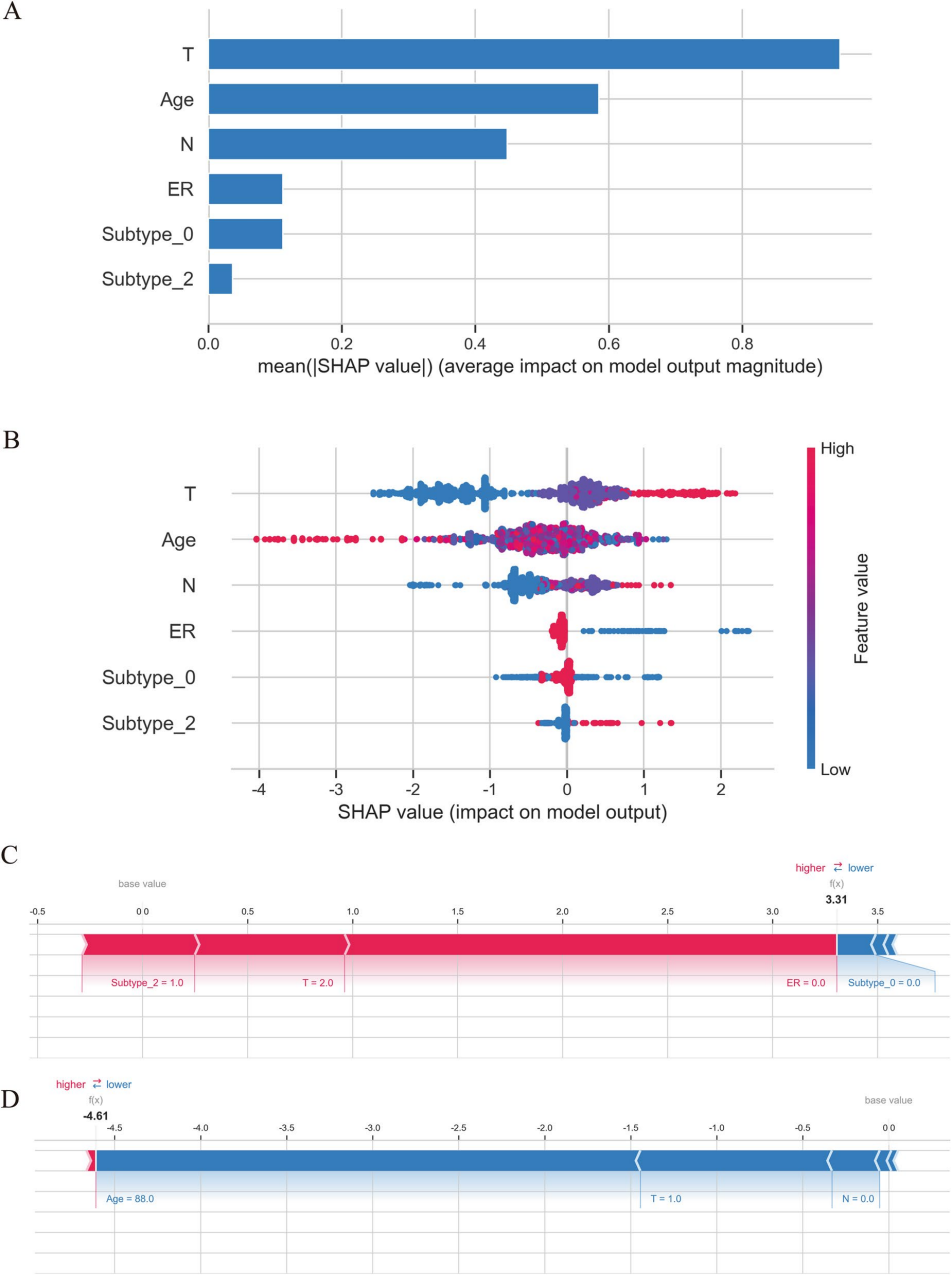
The XGB model’s interpretation. The importance ranking of the different variables according to the mean (∣SHAP value∣) (**A**); The importance ranking of different risk factors with stability and interpretation using the optimal model (**B**). The higher SHAP value of a feature is given, the higher risk of death the patient would have. The red part in feature value represents higher value. A classical sample with distant metastasis (**C**), and a classical sample without distant metastasis (**D**)

Based on the above results, the XGB model showed the best predictive ability. Therefore, the SHAP framework was introduced to interpret the model. Figure 5A illustrated all of the risk factors evaluated by the mean absolute SHAP value. T, age and N were the three most important variables. Figure 5B illustrated how the risk factors influence distant metastasis. The y-axis represented the value of risk factors, and the x-axis (SHAP value) represented the impact of risk factors on model output (distant metastasis). High T stage, lower age, high N stage, ER negative, and HR(-)/HER2(-)(subtype_2) increased the probability of distant metastasis.

The combination of different variables influenced the patient outcome. Therefore, to demonstrate the model's interpretability, we provided two classical samples: a distant metastasis patient with AJCC T2 stage and HR(-)/HER2(-) (Fig. 5C), and a patient with non-distant metastasis with AJCC.T1 and AJCC.N0 stage (Fig. 5D). The patient with distant metastasis had a high SHAP value (3.31) and a high prediction score (0.965); The patient without distant metastasis had a low SHAP value (-4.61) and a low prediction score (0.010).

### The application of the XGB model

**Fig. 6:**
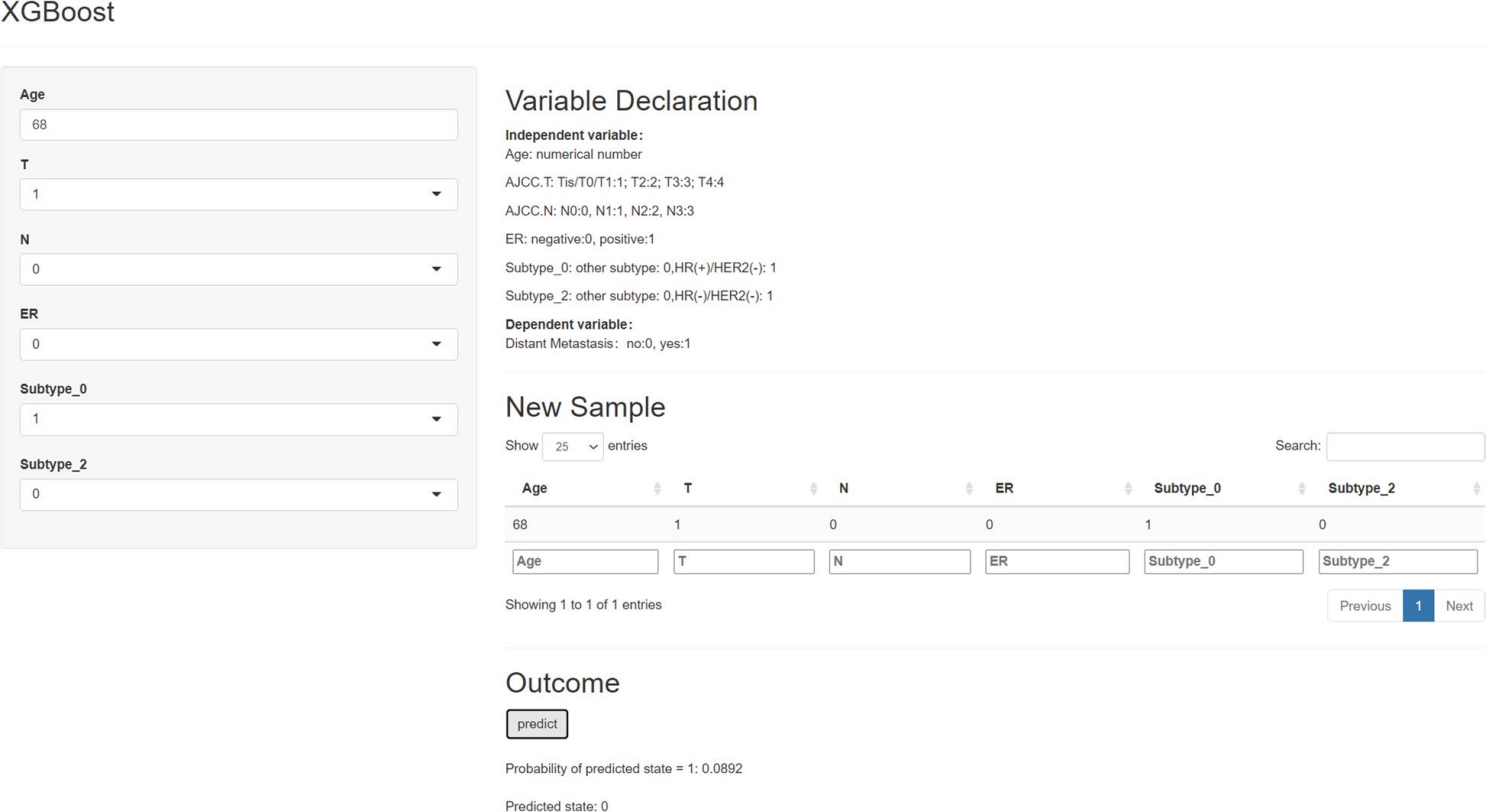
Screenshot of the Web APP based on XGBoost model, which is available at https://greenmood.shinyapps.io/male/

To make it easier for others to use this model, we developed a Web APP based on the XGB model. For example (Fig. 6), enter a patient's information into the model: age 68 years old, AJCC T1, AJCC N0, ER negative and HR( +)/HER2(-). Then, the model outputted a probability of distant metastasis was 0.0892, which indicated that this patient had a very low distant metastasis risk. The Web APP is available online (https://greenmood.shinyapps.io/male/).

## Discussion

Although MBC is rare, its incidence is gradually increasing. A previous study showed that MBC patients had a higher proportion of advanced disease than female breast cancer patients [26], which could be attributed to a lack of awareness and screening of breast cancer in MBC patients [27]. Therefore, it is necessary to discover and predict the risk of distant metastasis in a timely manner for MBC patients. This study demonstrated that predictive ability of the XGB model is better than that of other ML models and nomogram in predicting distant metastasis risk in male breast cancer patients. In addition, this model could also accurately predict the bone and lung metastasis risk. Through the SHAP value of each variable, the contribution and impact of each risk factor on mortality were intuitively demonstrated.

The clinicopathological characteristics of MBC are different from those of FBC. The results [25, 28] of the international MBC program demonstrated that the median age at diagnosis of MBC patients was 68.4 years old, and up to 99.3% patients were ER positive, while only 8.7% of patients were HER-2 positive. In this retrospective analysis from the SEER database of American and our hospital, similar clinicopathological characteristics of MBC were observed. The median age was 68.0 years old. Approximately half of the patients (1286, 54.7%) had a grade 2 cancer, as previously reported [29, 30]. Most patients belonged to the HR + /HER2- subtype (2038, 86.7%). Up to 97.5% patients were ER positive (99.1% in the validation set), and only 11.7% patients were HER2 positive (6.4% in the validation set). This study demonstrated that 168 (7.1%) patients had a distant metastasis and the two most common distant metastasis organs were bone and lung, which is also as previously reported [25].

In different international breast cancer guidelines, the standard of therapy for MBC is based on FBC [31, 32]. Although MBC patients could benefit from local treatment and systemic treatment, the prognosis of MBC is worse than that of FBC [26] because of the later stage at diagnosis or older age at diagnosis. In addition, MBC patients showed a higher risk of having contralateral breast cancer than FBC patients, which also increased the risk of death [33]. In addition, the delay in seeking medical treatment due to lack of knowledge or public education also leads to poor prognosis of MBC patients [34]. However, recent studies also found that MBC patients had a similar or a better prognosis than FBC patients after adjusting for some risk factors, such as age and stage [9, 10]. Therefore, early detection, early diagnosis and early treatment are very important to improve the prognosis of breast cancer. In clinical practice, we have noticed that many male patients refused professional breast examinations due to embarrassment or a lack of public education about MBC, which leads to a delay in getting medical attention. If we can develop a tool or model to predict the probability of mortality, it would be helpful to urge MBC patients to receive timely profession examination or treatment.

In recent years, ML models have also been widely applied to predict survival or lymph node metastasis of breast cancer [23, 35, 36]. However, it has not been used to predict the distant metastasis risk of MBC patients. In this research, we compared the predictive ability of four powerful ML algorithms, and XGB was the best model in predicting distant metastasis in MBC patients. The XGB model showed the largest mean AUC value in the tenfold CV (0.884) and the largest AUC value in the training (0.907), testing (0.0.827) and external validation (0.754) sets. These findings may be due to the unbalanced data (only 7.1% patients experienced distant metastasis) and limited sample size in the external validation set. However, we applied some statistical methods (such as SMOTE resampling) to address this problem. The calibration curves still demonstrated a slight deviation. However, the XGB model still presented a more perfect calibration curve and a better net benefit than the other three ML models with the smallest brier score (0.122) in the external validation set. In the future, a larger and balanced sample could present a better performance of XGB model. In addition, the XGB model also demonstrated a powerful ability to predict bone and lung metastasis in these three sets. Different from other ML model that lack of interpretability [37, 38], we introduced SHAP framework to interpret the “black box” of the XGB model. The feature importance of characteristics was intuitively observed through the summary plots based on the SHAP value. In addition, how a variable influences the outcome was intuitively shown by the SHAP value, and the force plots illustrated two classical personalized samples (Fig. 5).

To date, only one study has explored the relationship between clinicopathological characteristics and distant metastasis of MBC by nomogram [39]. However, the performance of the nomogram was poorer than that of our ML model in the training set (AUC: 0.822 vs 0.907) and lacked external validation, which also reduced the reliability and practicability of nomogram. Currently, an increasing number of ML models had been applied to the prediction of lymph node metastasis or survival state. However, it has not been used to predict distant metastasis in male breast cancer patients. In addition, no previous study has compared the ability of ML models and nomogram to predict distant metastasis in male breast cancer patients. Our previous study demonstrated that the XGB model had a better ability than the nomogram in predicting lymph node metastasis in breast invasive micropapillary carcinoma patients [23]. In this study, the results also showed that the XGB model had a better predictive ability than the nomogram in predicting M1 of MBC patients.

To make it easier for other researchers to use our model, we developed a public Web APP. After entering some necessary parameters, the user could obtain the probability of distant metastasis of an MBC patient. We believe that the model could urge MBC patients to receive standard treatment in time by telling them the probability of distant metastasis or help clinicians adjust the treatment plan in a timely manner.

This is the first study to develop, test and validate an ML model for the prediction of distant metastasis in MBC patients. Some limitations should also be noted. First, the data was extracted from SEER database of America, and our hospital is limited; more data from other regions will help the application of XGB model. Second, the information from the SEER database is finite, and using a cohort including more clinical and pathological characteristics (like AR status, Ki67 index, etc.) to train a model would help further improve the performance of ML model. Third, the TNM staging information from SEER database between 2010 to 2015 is blurry. Therefore, it is necessary to include pure pathological data to develop an ML model in the future.

## Conclusions

The XGB model is a better tool for the prediction of distant metastasis among MBC patients than other ML models and nomogram. It is also a powerful model for predicting bone and lung metastasis. The SHAP framework could effectively help clinicians intuitively understood how a variable influences the outcome of an MBC patient. The Web APP based on XGB model could help doctors adjust treatment plans or urge MBC patients to receive standard treatment in time.

## Supplementary Information

**Additional file 1.**

**Additional file 2.** 

**Additional file 3.** 

**Additional file 4.** 

## Data Availability

The data set (study population data) which is used for the construction of ML models generated and/or analyzed during the current study are available in the SEER database (https://seer.cancer.gov/). The data (external validation set) set which is used for the external validation of ML models used and/or analyzed during the current study are not publicly available due to the privacy policy of our hospital but are available from the corresponding author upon reasonable request. The Web App is developed based on the data of SEER database, and the relative codes and the detailed parameters are available from the corresponding author upon reasonable request.
