# Supplementary figures and images for: The prediction of distant metastasis risk for male breast cancer patients based on an interpretable machine learning model
Source: BMC Med Inform Decis Mak. 2023 Apr 21;23:74. doi: 10.1186/s12911-023-02166-8 (PMC10120176; doi:10.1186/s12911-023-02166-8)

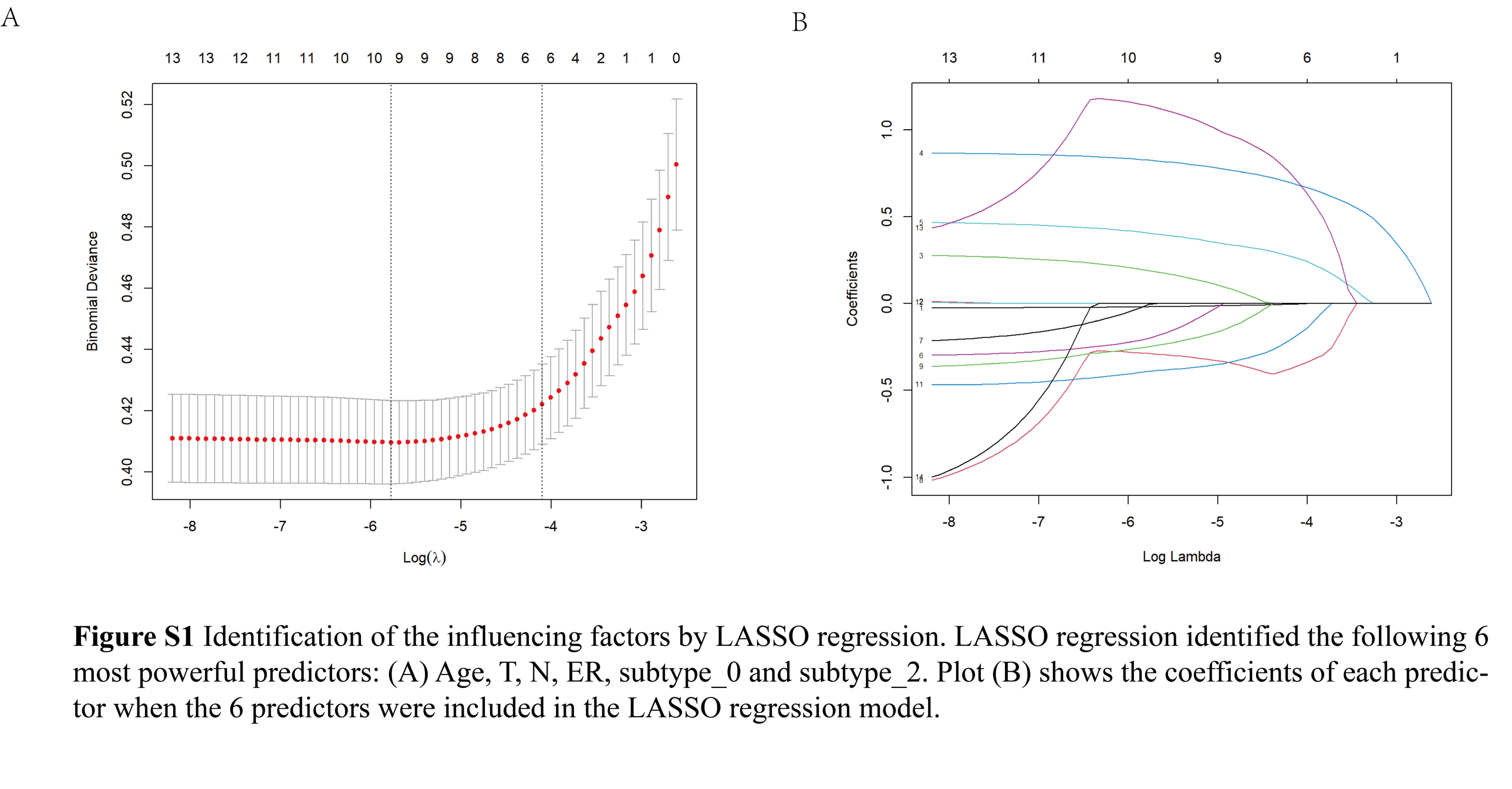

Supplement: Supplementary file 1 — Additional file 1. [file 12911_2023_2166_MOESM1_ESM.jpg]

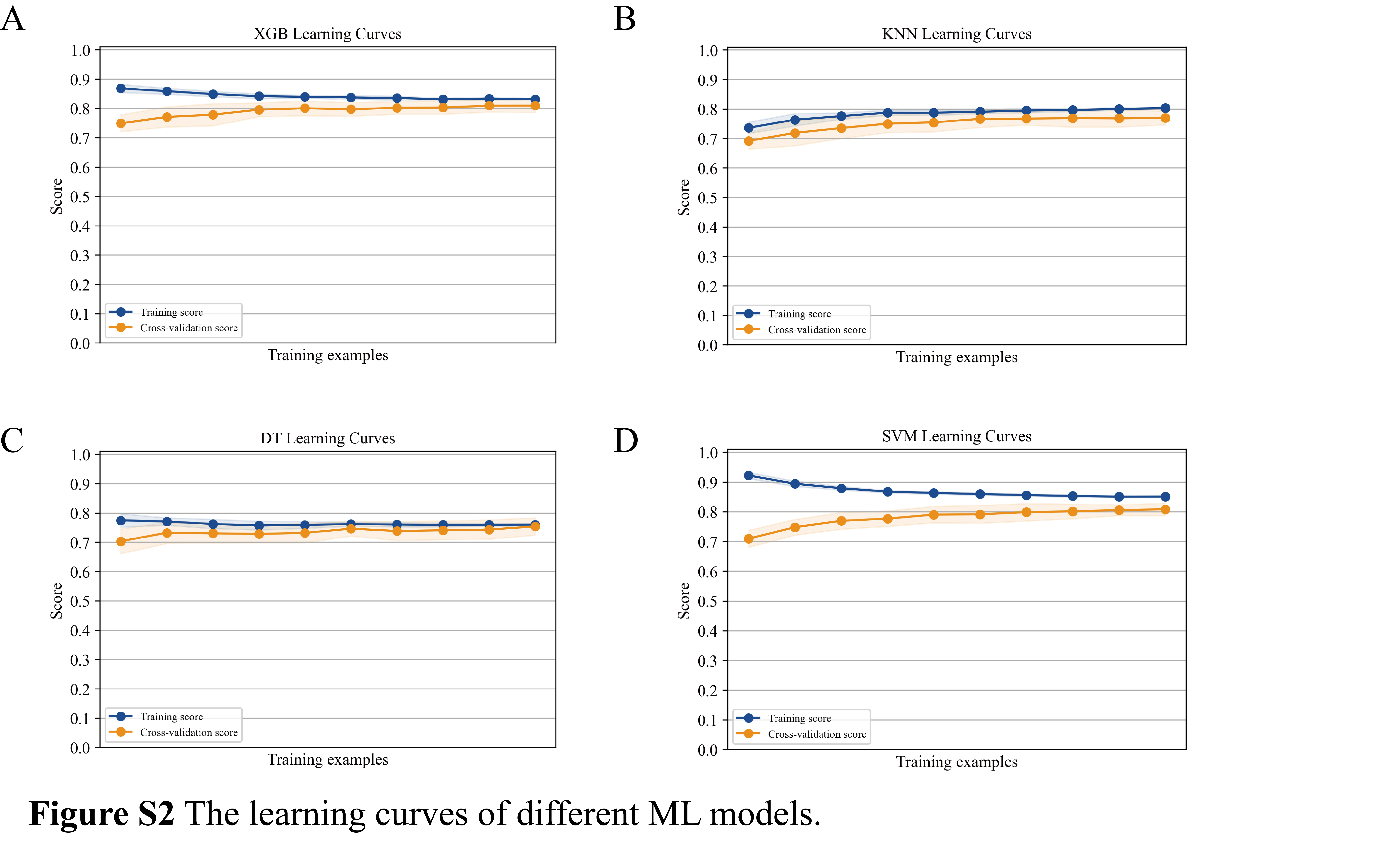

Supplement: Supplementary file 2 — Additional file 2. [file 12911_2023_2166_MOESM2_ESM.jpg]
