# Supplementary material for: The prediction of distant metastasis risk for male breast cancer patients based on an interpretable machine learning model
Source: BMC Med Inform Decis Mak. 2023 Apr 21;23:74. doi: 10.1186/s12911-023-02166-8 (PMC10120176; doi:10.1186/s12911-023-02166-8)
Supplement: Supplementary file 4 — Additional file 4. [file 12911_2023_2166_MOESM4_ESM.docx]

| **Table S1 Univariable and multivariable logistic regression analyses** | | | | |
| --- | --- | --- | --- | --- |
| **Variables** | **Univariable analysis** | | **Multivariable analysis** | |
|  | OR (95% CI) | p | OR (95% CI) | p |
| **Age** | 0.97 (0.96-0.99) | <0.001 | 0.97 (0.96-0.99) | **0.003** |
| **Laterality** |  |  |  |  |
| left | 1(reference) |  |  |  |
| right | 0.93 (0.63-1.38) | 0.713 |  |  |
| **Grade** |  |  |  |  |
| 1 | 1(reference) |  | 1(reference) |  |
| 2 | 3.6 (1.11-11.69) | 0.033 | 3.24 (0.97-10.87) | 0.057 |
| 3/4 | 6.56 (2.03-21.2) | 0.002 | 4.17 (1.24-14.04) | **0.021** |
| **AJCC.T** |  |  |  |  |
| T0/Tis/T1/T2 | 1(reference) |  | 1(reference) |  |
| T3/T4/TX | 6.48 (4.27-9.83) | <0.001 | 5.12 (3.27-8.03) | **<0.001** |
| **AJCC.N** |  |  |  |  |
| N0 | 1(reference) |  | 1(reference) |  |
| N1 | 3.63 (2.27-5.83) | <0.001 | 3.05 (1.82-5.11) | **<0.001** |
| N2 | 2.73 (1.33-5.61) | 0.006 | 2.23 (1.01-4.92) | **0.048** |
| N3/NX | 8.13 (4.29-15.4) | <0.001 | 4.8 (2.32-9.93) | **<0.001** |
| **Radiotherapy** |  |  |  |  |
| no | 1(reference) |  |  |  |
| yes | 1.39 (0.92-2.11) | 0.120 |  |  |
| **Chemotherapy** |  |  |  |  |
| no | 1(reference) |  | 1(reference) |  |
| yes | 1.8 (1.22-2.66) | 0.003 | 0.71 (0.44-1.16) | 0.171 |
| **Subtype*** |  |  |  |  |
| HR(+)/HER2(-) | 1(reference) |  |  |  |
| HR(+)/HER2(+) | 2.36 (1.41-3.94) | 0.001 |  |  |
| HR(-)/HER2(-) | 8.53 (3.71-19.64) | <0.001 |  |  |
| HR(-)/HER2(+) | 5.12 (1.38-19) | 0.015 |  |  |
| **ER** |  |  |  |  |
| negative | 1(reference) |  | 1(reference) |  |
| positive | 0.15 (0.08-0.3) | <0.001 | 0.19 (0.07-0.52) | **0.001** |
| **PR** |  |  |  |  |
| negative | 1(reference) |  | 1(reference) |  |
| positive | 0.34 (0.21-0.57) | <0.001 | 0.73 (0.35-1.51) | 0.393 |
| **HER2** |  |  |  |  |
| negative | 1(reference) |  | 1(reference) |  |
| positive | 2.29 (1.41-3.71) | 0.001 | 1.45 (0.83-2.52) | 0.188 |

*Subtype was excluded from multivariate analysis because of a VIF value ＞5.
